# Low-level light treatment ameliorates immune thrombocytopenia

**DOI:** 10.1038/srep38238

**Published:** 2016-11-30

**Authors:** Jingke Yang, Qi Zhang, Peiyu Li, Tingting Dong, Mei X. Wu

**Affiliations:** 1 Wellman Center for Photomedicine, Massachusetts General Hospital, Department of Dermatology, Harvard Medical School, Boston, MA 02114, USA; 2 Affiliated faculty member of the Harvard-MIT Division of Health Sciences and Technology, Cambridge, MA 02115, USA.

## Abstract

Immune thrombocytopenia (ITP) is an immune-mediated acquired bleeding disorder characterized by abnormally low platelet counts. We reported here the ability of low-level light treatment (LLLT) to alleviate ITP in mice. The treatment is based on noninvasive whole body illumination 30 min a day for a few consecutive days by near infrared light (830 nm) transmitted by an array of light-emitting diodes (LEDs). LLLT significantly lifted the nadir of platelet counts and restored tail bleeding time when applied to two passive ITP models induced by anti-CD41 antibody. The anti-platelet antibody hindered megakaryocyte differentiation from the progenitors, impaired proplatelet and platelet formation, and induced apoptosis of platelets. These adverse effects of anti-CD41 antibody were all mitigated by LLLT to varying degrees, owing to its ability to enhance mitochondrial biogenesis and activity in megakaryocytes and preserve mitochondrial functions in platelets in the presence of the antibody. The observations argue not only for contribution of mitochondrial stress to the pathology of ITP, but also clinical potentials of LLLT as a safe, simple, and cost-effective modality of ITP.

Immune thrombocytopenia (ITP) is an autoimmune hemorrhagic disorder, characterized by a transient or persistent decline of circulating platelets and the absence of other conditions known to induce thrombocytopenia. Its overall incidence is between 1.9 and 6.4 per 10^5^ children and 3.3 per 10^5^ adults annually^1^. Signs and symptoms of ITP vary widely with patients. Most of them have either no symptoms or minimal bruising, whereas others may experience serious bleeding, including extensive skin, mucosal, gastrointestinal or intracranial hemorrhage^2^. ITP patients are primarily treated by corticosteroids as a first-line therapy with response rates of 70–90%. However, long-term use of corticosteroids is not recommended due to side effects such as gastrointestinal distress and osteoporosis^2^,^3^. The majority of patients treated with corticosteroid relapses during drug tapering or after withdrawal, and thus requires further therapy^2^. One of the standard second-line therapies is splenectomy, which offers approximately 60% long-term stable response rate but with an increased risk for postoperative complications^4^,^5^. In recent years, antibody against B cells named rituximab and thrombopoietin (TPO) receptor agonists like eltrombopag and romiplostim serve as second-line options with good tolerance and promising response rates^6^,^7^. Spleen tyrosine kinase inhibitors such as fostamatinib that is in phase III clinical trials might also offer another therapy for ITP. However, rituximab sometimes increased susceptibility to infections and neutropenia^8^,^9^. Clinical study showed that long-term treatment with eltrombopag or romiplostim could trigger some adverse effects, including liver enzyme elevation, thrombolic events and increased bone marrow reticulin^6^,^7^. Furthermore, these second-line drugs are all very expensive. We aimed at development of a simple, safe, and cost-effective alternative to manage ITP.

Low-level light refers to red to near infrared lasers with a wavelength of 600–1,100 nm, an output power of 1–500 mW, and relatively low energy densities (0.04–50 J/cm^2^), in either a continuous wave or pulsed mode. It has been used in the clinics for decades in wound healing, tissue repair, pain relief, and inflammation reduction with a long safety record^10^,^11^,^12^,^13^. Mitochondrial cytochrome *c* oxidase is currently thought to be one of the major photoacceptors for the initial effects of low-level light^14^. Several investigators, including us, have shown that low-level light treatment (LLLT) modulates ATP production, reactive oxygen species (ROS) formation, apoptosis, cellular metabolic process, and signaling transduction pathways secondarily to more sufficient function of mitochondria under various conditions of stress^15^,^16^,^17^. Recently, we discovered that 810-nm continuous wave diode laser at 30 J/cm^2^ could penetrate into mouse bone marrow noninvasively without incurring any significant heat^18^. Noninvasive whole body illuminations with the LLLT cured acute thrombocytopenia induced by irradiation, chemotherapeutic drug, or anti-CD41 antibody much faster than sham-light treatment^18^. We found that LLLT bolstered mitochondrial biogenesis predominantly in polyploid megakaryocytes (MKs), increasing platelet production both *in vivo* and *in vitro*^18^. In the current study, we apply an array of light-emitting diodes (LEDs), instead of diode laser, to two passive ITP mouse models to gain similar results. In comparison with diode laser, LED is safer and more convenient and cost-effective. Moreover, an LED array can illuminate a relative large area of the body in a hands-free manner, in sharp contrast to a point-by-point application of a laser system^19^. LED illumination appears as effectively as a diode laser in ITP amelioration.

## Results

### LLLT ameliorates ITP

**Figure 1:**
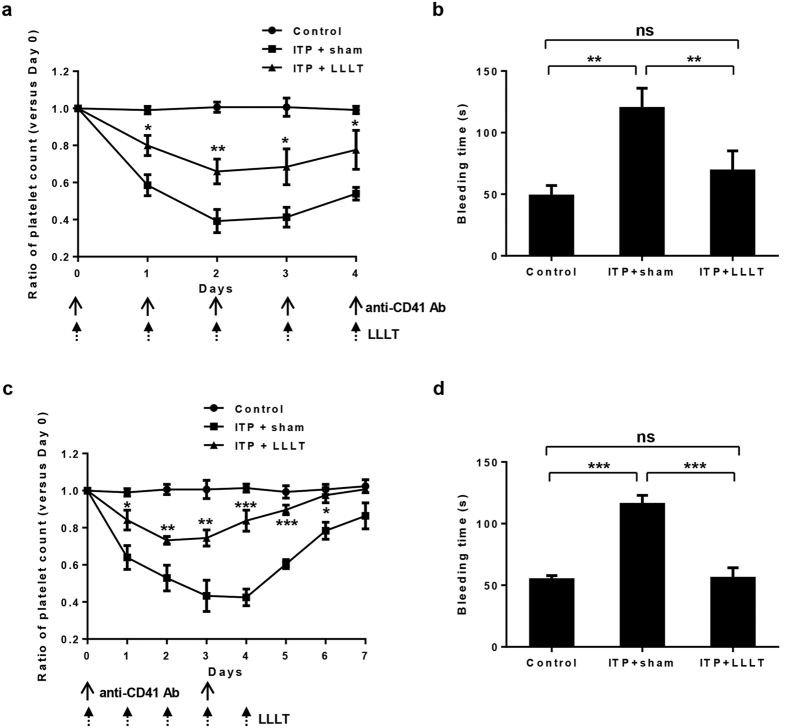
LLLT ameliorates ITP. Mice were administered with anti-CD41 antibody (ITP) at either 0.1 mg/kg daily (**a,b**) or twice, one on day 0 and the other on day 3 each at 0.5 mg/kg (**c,d**). Control mice received PBS only. Noninvasive whole body LLLT at 36 J/cm^2^ was administered daily at 4 hr after each antibody injection from day 0 to day 4. Platelet counts (**a,c**) were enumerated before each antibody injection. Tail bleeding time (**b**,**d**) was assessed on day 2 (**b**) or 4 (**d**) after the first antibody injection. All data represent mean ± SEM; n = 12; ns, no significance; and *P < 0.05, **P < 0.01, ***P < 0.001 compared in the presence or absence of LLLT or between indicated groups.

To investigate thrombopoietic effects of LLLT, we used a passive ITP mouse model by daily injecting anti-CD41 antibody at 0.1 mg/kg body weight. The mice were then treated with either sham light or 830-nm LED at 36 J/cm^2^ in 4 hours after anti-CD41 antibody injection and the similar LLLT continued once a day up to 5 days. Platelet counts were enumerated daily before each antibody injection. As shown in Fig. 1a, a single dose of anti-CD41 antibody diminished platelet counts by 40% in ITP+sham group compared with the control group. LLLT lifted the nadir effectively by only two treatments (P < 0.01) in ITP+LLLT group when compared to ITP+sham group. Continuous LLLT daily greatly prevented a loss of platelets and sustained platelet counts above 60% of the control, whereas the mice remained severely thrombocytopenic in ITP+sham group, with platelet counts below 50% of the control (P < 0.01 on day 2; P < 0.05 on day 3; and P < 0.05 on day 4). Moreover, bleeding time in ITP+sham group was lengthened to 120 sec from 50 sec in the control group on day 2 (P < 0.01, Fig. 1b), and LLLT shortened the bleeding time by 50%, leading to a nearly normal level in ITP+LLLT group (P < 0.01, Fig. 1b). We then extended our investigation to another ITP model by administering anti-CD41 antibody twice one on day 0 and the other on day 3 each at 0.5 mg/kg body weight. The mice were treated with sham light or LLLT daily from day 0 to day 4 (Fig. 1c). The antibody once again lowered platelet counts significantly one day after, and platelet counts reached the nadir in 3~4 days with an ~60% decline and remained significantly lower up to day 6 in ITP+sham group. The decline of platelet counts was however largely prevented by daily LLLT for 5 days, concurrent with normalization of bleeding time in the mice on day 4 in ITP+LLLT group (Fig. 1d). The result confirms that LLLT restores not only platelet counts but also platelet function in mice receiving anti-CD41 antibody.

### LLLT partially reverses antibody-mediated inhibition of MK differentiation

**Figure 2:**
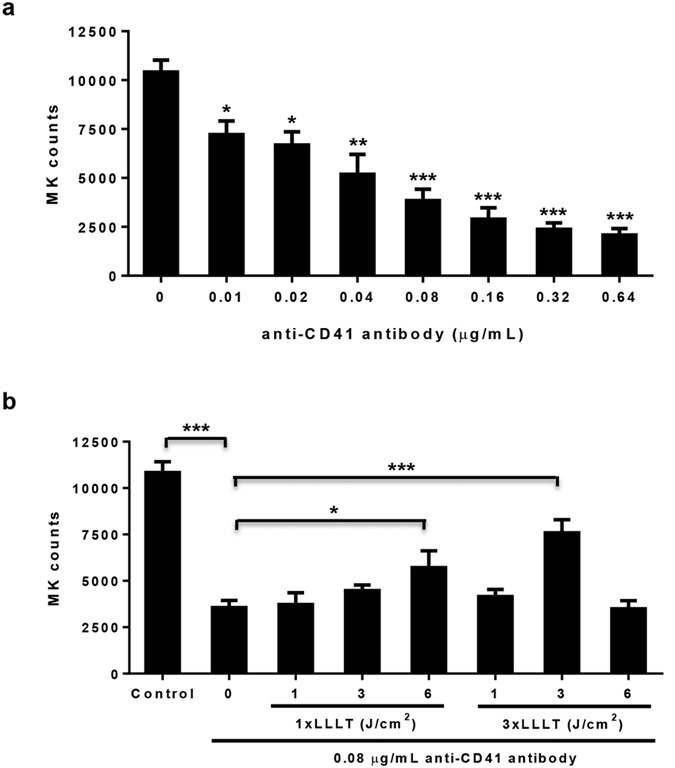
LLLT rescues MK differentiation in the presence of anti-CD41 antibody. (**a**) Bone marrow nucleated cells were differentiated in MK medium containing indicated concentrations of anti-CD41 antibody. The number of MKs was quantified 72 hr later by flow cytometry. (**b**) Bone marrow nucleated cells were cultured for 2 hr in MK medium containing 0.08 μg/mL anti-CD41 antibody, after which LLLT at indicated energy density was given either once (1 x LLLT) or once a day for 3 consecutive days (3 x LLLT). On day 3, the number of MKs was measured by flow cytometry as (**c**). All data represent mean ± SEM; n = 6, *P < 0.05, **P < 0.01, and ***P < 0.001 compared in the presence or absence of LLLT or between indicated groups.

To address the mechanism underlying thrombopoietic effect of LLLT, mouse bone marrow nucleated cells were differentiated for 72 hr in the presence of an increasing concentration of anti-CD41 antibody in serum-free expansion medium supplemented with 100 ng/mL TPO, called MK medium hereafter. Anti-CD41 antibody significantly lowered the number of MKs in a dose-dependent manner (Fig. 2a), in agreement with hampering MK differentiation by the antibody. The number of MKs was reduced by 65% in the presence of 0.08 μg/mL anti-CD41 antibody (P < 0.001, Fig. 2a), and this antibody concentration was used in subsequent investigation of LLLT efficacy. Exposure of the cell culture to a single dose of LLLT (1 x LLLT) at either 1 or 3 J/cm^2^ at 2 hr after antibody addition exhibited little effect on MK differentiation (Fig. 2b), while increasing the energy density to 6 J/cm^2^ gave rise to only modest effects (P < 0.05). On the contrary, three doses of LLLT (3 x LLLT) at 3 J/cm^2^ administered to the cell culture once a day for 3 consecutive days starting 2 hr after antibody addition displayed the most significant effect on MK differentiation, increasing the MK number by 110% in the presence compared to the absence of LLLT (P < 0.001, Fig. 2b), although similar three doses of LLLT were not effective if laser energy was lower (1 J/cm^2^) or higher (6 J/cm^2^). This biphasic dose-response of LLLT was also reported previously in LLLT-mediated ATP synthesis and recovery of traumatic brain injury^20^,^21^. The finding that antibody-mediated inhibition of MK differentiation can be significantly reversed only after multiple doses of LLLT may be due to its accumulative effect on a specific differentiation stage of MKs, which is supposed to be continuously formed throughout the 3-day differentiation culture. The ability of LLLT to partially reverse the antibody-mediated inhibition of MK differentiation may contribute, at least in part, to its mitigation of ITP *in vivo* (Fig. 1).

### LLLT improves proplatelet and platelet formation in the presence of anti-CD41 antibody

**Figure 3:**
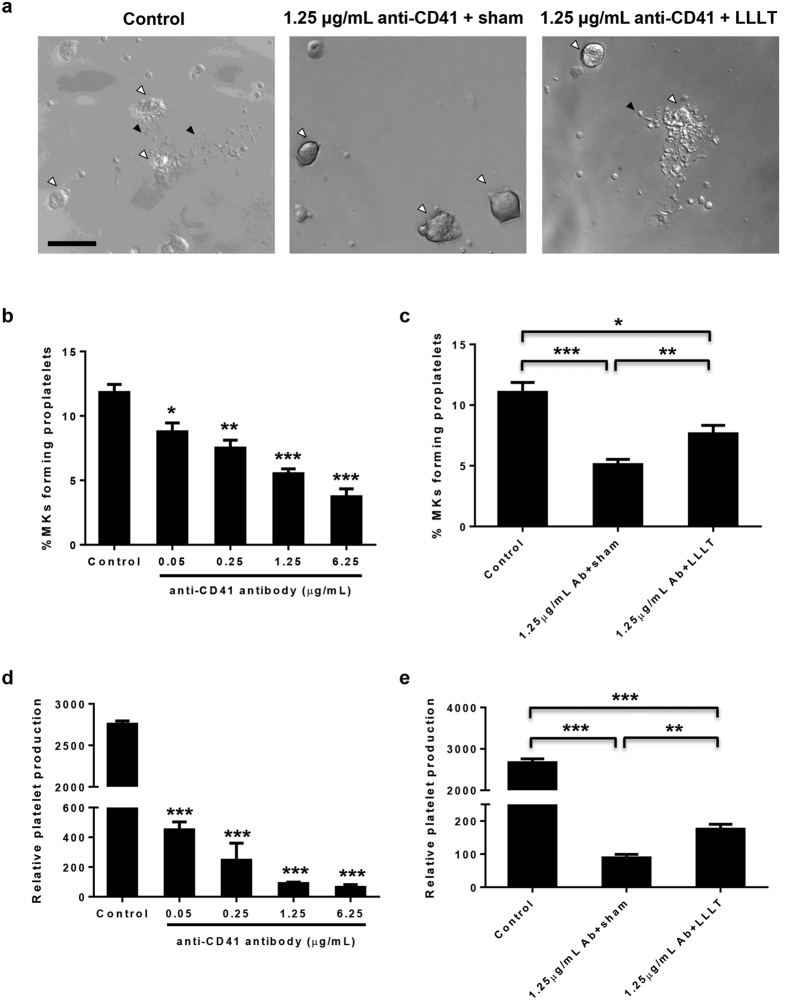
LLLT partially reverses antibody-mediated hindrance of proplatelet and platelet formation. Mature MKs were sorted and differentiated in MK medium with or without indicated concentrations of anti-CD41 antibody and LLLT was given 2 hr later. (**a**) Representative images of proplatelet formation at 24 hr after initial culture. Filled triangles represent one of many protrusions on proplatelet shafts and unfilled triangles indicate the nucleus. Scale bar, 50 μm. The percentages of MKs forming proplatelets were determined in the presence of indicated concentrations of anti-CD41 antibody without LLLT (**b**) or with LLLT (**c**). (**d**) The number of platelets derived from 1 × 10^4^ MKs was estimated after the cells were differentiated in MK medium for 3 days in the presence of indicated concentrations of anti-CD41 antibody. Antibody-mediated inhibition of platelet generation was partially reversed by LLLT (**e**). All data represent mean ± SEM; n = 6, *P < 0.05, **P < 0.01, and ***P < 0.001 compared with control groups or in the presence or absence of LLLT.

Apart from partial reversal of antibody-mediated inhibition of MK differentiation, LLLT appeared to favorably affect the final stages of platelet generation in the presence of anti-CD41 antibody as well. In a 24 hr differentiation culture of MKs, the cells converted their entire cytoplasm into many long protrusions and branches of proplatelets forming a complex network or blossom-like cell, which were readily seen under a phase contrast microscope in the absence of anti-CD41 antibody (Fig. 3a, left panel), but the complex networks of proplatelet-forming MKs were seldom seen in the presence of the antibody (Fig. 3a, middle panel), corroborating severe hindrance of proplatelet formation by the antibody. Percentages of proplatelet-forming MKs declined with a rising concentration of anti-CD41 antibody from 0.05 to 6.25 μg/mL in a dose-dependent manner (Fig. 3b). While anti-CD41 antibody at 1.25 μg/mL caused about 55% reduction in the number of proplatelet-forming MKs (P < 0.001, Fig. 3c), a single dose of LLLT at 3 J/cm^2^ given to the cell culture at 2 hr after antibody addition cut the loss by half (Fig. 3a, right panel; and P < 0.01, Fig. 3c). Increasing doses or energy density per dose of LLLT did not yield better result in this one-day culture (data not shown). A reduced number of proplatelet-forming MKs was proportionally correlated with decreased platelet production in a 3-day differentiation culture. Hence, an increasing anti-CD41 antibody from 0.05 to 6.25 μg/mL significantly diminished platelet production from 6 to 50 fold (Fig. 3d), whereas LLLT restored platelet production by 2-fold in the presence of 1.25 μg/mL anti-CD41 antibody (P < 0.01, Fig. 3e), although it was still much lower than antibody-free controls (p < 0.001, Fig. 3e). These results hint that anti-CD41 antibody impairs, but LLLT partially restores, generation of proplatelets and platelets in ITP.

### LLLT enhances mitochondrial function and biogenesis in antibody-treated MKs

**Figure 4:**
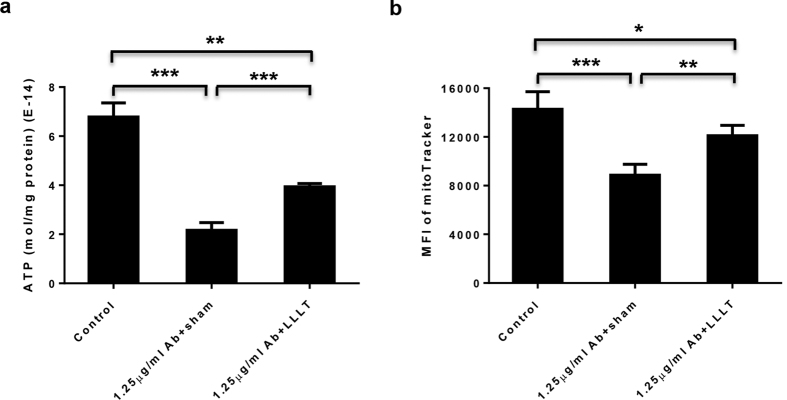
LLLT enhances mitochondrial function and biogenesis in antibody-treated MKs. Sorted MKs were differentiated in MK medium supplemented with 1.25 μg/mL anti-CD41 antibody, followed 2 hr later by LLLT or sham light treatment. (**a**) ATP was measured in 1 × 10^5^ MKs at 30 min post-LLLT. (**b**) Mitochondrial mass was determined 24 hr post-LLLT by staining with MitoTracker followed by flow cytometric analysis. All data represent mean ± SEM; n = 6, *P < 0.05, **P < 0.01, and ***P < 0.001 compared in the presence or absence of antibody or LLLT.

Our recent study showed that LLLT bolstered ATP production and mitochondrial biogenesis in MKs^18^. Whether or not this held true in the presence of anti-CD41 antibody remained unknown. To investigate this, MKs were differentiated in MK medium supplemented with 1.25 μg/mL anti-CD41 antibody. The differentiation cell cultures were subject to sham light or a single dose of LLLT at 3 J/cm^2^ at 2 hr after antibody incubation, followed by measuring cellular ATP levels in 30 min. ATP production declined drastically in antibody-treated MKs in comparison with control cells (P < 0.001, Fig. 4a). A similar declining trend was also evidenced in mitochondrial mass, as measured by MitoTracker staining, at 24 hr after initial differentiation culture (P < 0.001, Fig. 4b). Both these antibody-mediated impairments were partially but significantly prevented by a single dose of LLLT (P < 0.01, Fig. 4a,b).

### LLLT prevents platelet apoptosis and prolongs its lifespan

**Figure 5:**
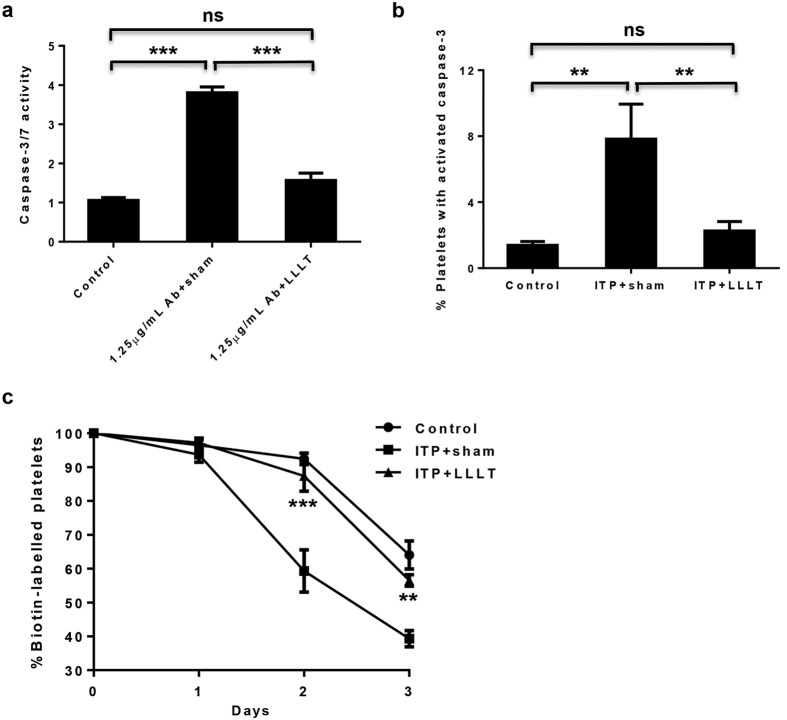
LLLT inhibits platelet apoptosis and prolongs its lifespan. (**a**) Platelets were prepared from blood samples of mice and incubated for 2 hr in MK medium containing 1.25 μg/mL anti-CD41 antibody before treated with LLLT. Caspase-3/7 activity in platelets was measured at 6 hr post-LLLT using Caspase-Glo 3/7 reagents. (**b**) The passive mouse ITP model was induced and daily LLLT was administered as Fig. 1a. The percentage of platelets expressing activated caspase-3 was assessed by FAM-DEVD-FMK fluorescence in platelets prepared from mice 2 days after treated with or without two doses of anti-CD41 antibody and LLLT. (**c**) Platelets were biotinylated *in vivo*, followed by ITP induction and LLLT as Fig. 1a. The percentage of biotin-labeled platelets in the circulation was determined by flow cytometry before each antibody injection. All data represent mean ± SEM; n = 12; ns, no significance; and *P < 0.05, **P < 0.01, ***P < 0.001 compared in the presence or absence of LLLT or anti-CD41 antibody.

In light of a well-documented role for mitochondrial activity in the lifespan of platelets^22^, we next determined whether LLLT could protect platelets from apoptosis and prolong their lifespan, an yet another factor contributing to mitigation of ITP by LLLT seen in Fig. 1. To this end, platelets were prepared from mouse blood samples, cultured in MK medium, and treated with or without anti-CD41 antibody for 2 hr before exposure of the platelets to LLLT. Measurement of the caspase-3/7 activity in the platelets revealed a 3.6-fold increase in caspase-3/7 activity, on average, in the presence compared to the absence of anti-CD41 antibody (P < 0.001, Fig. 5a). The antibody-induced activation of caspase3/7 was profoundly blunted by LLLT when measured 6 hr post-LLLT (P < 0.001, Fig. 5a). This *ex vivo* observation was subsequently corroborated *in vivo* in the passive mouse ITP model treated with LLLT daily at 36 J/cm^2^ as Fig. 1a. As shown in Fig. 5b, daily injection of anti-CD41 antibody led to about 5.6-fold increases in the percentage of platelets expressing activated caspase-3 (p < 0.01, Fig. 5b), when platelets were isolated from the mice on day 2 after antibody injection and assayed for caspase-3 activation. However, the proportion of apoptotic platelets declined to a near normal level by two doses of noninvasive whole body LLLT of the mice (P < 0.01, Fig. 5b). Since platelet apoptosis is correlated with platelet lifespan and clearance^23^, we monitored circulating platelets in these mice after injection of a biotinylation agent as previously described^24^, which is a common assay for monitoring the lifespan of platelets in the circulation. In comparison with control mice, the percentage of biotin-labeled platelets was decreased precipitously on day 2 and day 3 and remained below a 50% level on day 3 in ITP + sham mice (Fig. 5c). In contrast, LLLT was able to sustain biotin-labeled platelets at normal levels, increasing biotin-labeled platelets to 87% from 60% on day 2 (P < 0.001, Fig. 5c) and to 56% from 40% on day 3 (P < 0.01, Fig. 5c). These results suggest that LLLT prevents platelets from apoptosis and prolongs their lifespan in the presence of anti-platelet antibody.

## Discussion

The current investigation demonstrates the ability of LLLT to alleviate thrombocytopenia in two ITP mouse models^25^, which are different from the one we recently tested^18^. While both studies consistently show a potential of LLLT to be a novel modality to treat thrombocytopenia, the current study is tailored specifically for ITP, especially how LLLT can correct the adverse effect of anti-platelet antibody on platelet biogenesis and the survival of platelets. Anti-CD41 antibody appears to impair multiple discernible differentiation steps of platelet biogenesis, including MK differentiation from the progenitors, proplatelet formation, platelet generation, and the survival of platelets. Strikingly, LLLT is able to ameliorate all of these defects caused by anti-CD41 antibody at varying degrees through enhancing mitochondrial activity and/or biogenesis, resulting in ITP alleviation and restoration of tail bleeding time in antibody-treated mice. The study underscores contribution of mitochondrial stress either directly or indirectly to the pathology of ITP, which may open novel avenues for ITP management, for instance, by using mitochondrion-promoting drugs.

A loss of immune tolerance to self-antigens is believed resulting in development of various autoantibodies against platelets, MKs, and MK progenitors^26^. These autoantibodies impair MK differentiation and platelet generation and destroy platelets, leading to a low platelet count. The impaired megakaryocytopoiesis and platelet production in ITP pathogenesis have been demonstrated in several animal models and MK morphologic studies^27^,^28^. *In vitro* cell culture studies have shown that plasma of ITP patients hinders MK differentiation^29^,^30^,^31^ and in some instances also inhibits proplatelet formation^32^ because the plasma contains autoantibodies directed at platelets. Similarly, we found that anti-CD41 antibody hampered MK differentiation, proplatelet formation, and platelet generation in a dose-dependent manner (Fig. 3). The investigation provides additional evidence that hindrance of the final stages of MK differentiation by autoantibodies contributes to immunopathological mechanisms of ITP^32^. Two previous studies showed that anti-platelet autoantibodies isolated from chronic ITP patients perturbed MK polyploidy apart from MK maturation^30^,^31^. This is apparently not the case in our investigation because we did not observe any significant reduction of polyploid MKs when bone marrow cells were differentiated into MKs for 3 days in MK medium supplemented with anti-CD41 antibody (data not shown), probably owing to different antibodies involved. It is also possible that the discrepancy results from a shorter differentiation time (3 days) in our study, as opposed to a 15-day liquid MK differentiation system in those two studies^30^,^31^. Nevertheless, our result is consistent with another study using umbilical cord blood cells cultured for 8 days, showing the ploidy distributions of MKs were not significant different in cultures containing either control or ITP plasma^29^. Although adverse effects of anti-CD41 antibody on discernible stages of MK differentiation, proplatelet and platelet formation and the survival of platelets are well described, the underlying mechanism remains poorly understood.

Our finding that LLLT could significantly sustain MK maturation and proplatelet and platelet generation in the presence of anti-CD41 antibody suggests direct or indirect contribution of antibody-induced mitochondrial stress to the pathology of ITP, in light of the well-documented benefits of LLLT to mitochondria. LLLT has been shown to preserve mitochondrial function and increase ATP synthesis in various cells under stress^12^,^13^,^33^. Moreover, we recently demonstrated that LLLT protected mitochondrial function in irradiated MKs, facilitating thrombopoiesis after irradiation in mice^18^. MK maturation and proplatelet formation are highly energy-consuming processes and mitochondria are crucial in these differentiation processes as we showed that the yield of platelet production was correlated proportionally with the number of mitochondria or the level of ATP production in the cells^18^. An importance of mitochondria in the final stage of platelet biogenesis is also consistent with migration of MKs from low-oxygen niches in the bones toward the sinusoidal blood vessels during proplatelet formation and platelet release from MKs because a high level of oxygen around the sinusoidal blood vessels warrants higher mitochondrial oxidative phosphorylation^34^. On the contrary, inadequate ATP production in MKs lacking immediate early responsive gene X-1 (IEX-1) hindered proplatelet formation^18^. In accordance with this, anti-CD41 antibody severely blunts ATP production and/or mitochondrial biogenesis (Fig. 4), which correlates well with hindrance of MK differentiation and platelet biogenesis by the antibody. The observations argue not only for mitochondrial stress as one of the etiologies of ITP but also for the mechanism underlying LLLT-mediated alleviation of ITP.

Apart from inhibition of MK differentiation and platelet formation, anti-platelet antibody appears to induce apoptosis of platelets, which may be another factor contributing to the immunopathology of ITP^23^,^26^,^35^. Anti-platelet autoantibody has been shown to increase mitochondrial inner membrane potential (∆ψ_m_) depolarization, caspase-3,8,9 activation and phosphatidylserine exposure in platelets^23^,^35^, indicating a mitochondrion-dependent apoptosis pathway involved. In support of a role for platelet apoptosis in ITP pathogenesis, caspase-3 activation is greatly elevated in platelets by anti-CD41 antibody, shortening the lifespan of circulating platelets. On the contrary, LLLT significantly inhibits platelet apoptosis, and prolongs platelet lifespan, a finding that is not reported in our previous study^18^. The mechanism underlying LLLT-mediated protection against apoptosis of platelets is likely associated with the ability of LLLT to protect mitochondrial functions in the presence of anti-CD41 antibody.

LLLT has been a therapeutic approach for analgesics, anti-inflammation and wound healing in the clinics for 30 years with a long recorded safety. Although the current study primarily targets thrombocytopenia induced by anti-CD41 antibody, LLLT-mediated enhancement of platelet production should be extended to ITP caused by other etiologies, such as anti-CD42b antibody or pathogenic T/B cell reactivity^29^,^36^, as well as to those acquired thrombocytopenia that are not mediated by an immune mechanism including HIV, chemotherapy, and irradiation-induced thrombocytopenia^18^.

## Methods

### Mice

C57BL/6 mice at 6–8 weeks of age were purchased from Charles River Laboratories. The mice were maintained in a 12-hour light/dark cycle. The animal protocol was approved by the subcommittee on Research Animal Care of the Massachusetts General Hospital. All animal experiments in this study were performed according to the National Institutes of Health guidelines for the Care and Use of Laboratory Animals.

### ITP induction

Mice were intraperitoneally injected with anti-CD41 antibody (BD Bioscience) either daily at 0.1 mg/kg body weight in PBS or on day 0 and 3 only each at 0.5 mg/kg body weight. Control mice were intraperitoneally injected with 400 μL PBS in parallel. Platelet counts were analyzed on a HemaTrue veterinary hematology analyzer (Heska Corporation) with 50 μL whole blood collected from the retro-orbital venous plexus into EDTA-coated microtainer tubes (BD Bioscience)

### Low-level light treatment

LLLT was performed by an array of near infrared LEDs at 830 nm (Photo Therapeutics, Inc). For whole body illumination of a mouse, the hair-removed mouse was subject to LED irradiation at 4 hr after anti-CD41 antibody injection. The average irradiance of LED was 20 mW/cm^2^ and the total exposure time was 30 minutes to achieve an energy density of 36 J/cm^2^. For *in vitro* studies, MK or platelet cultures were exposed to LED at 2 hr after anti-CD41 antibody incubation, with an average irradiance of 20 mW/cm^2^ and different energy densities specified in each experiment. The sham light was administered similarly with a small soft white LED light bulb (3 W, A15) from General Electric.

### Bleeding time analysis

Tail bleeding time was determined as previously described^24^. Briefly, the tail was transected at 5 mm from the tip while the mouse was strained in a restrainer (Braintree Scientific). The bleeding tail was immersed in a 15-ml test tube containing 37 °C pre-warmed PBS. Then the time to cessation was measured.

### MK differentiation from bone marrow cells

Bone marrow cells were flushed from femur and tibia of the mice by Hanks’ balanced salt solution (HBS, Sigma) supplemented with 100 U/mL penicillin-streptomycin (Life Technologies), and filtered through a 100-μm strainer. After removal of red blood cells using ACK lysing buffer (Quality Biological), the cells were seeded in 6-well plates at a concentration of 1 × 10^6^ cells/mL in StemSpan serum-free expansion medium (STEMCELL Technologies) supplemented with 100 ng/ml TPO and differentiated for indicated days.

### Flow cytometric analysis and cell sorting

To sort MKs, mouse bone marrow cells were incubated with fluorescein isothiocyanate (FITC)-conjugated anti-mouse CD41 antibody (Biolegend) for 20 min on ice. Mature MKs were sorted using FACSAria (BD Bioscience) based on CD41 expression and forward/side scatter^high^. To quantify platelets produced by MKs, platelets were collected from day-3 differentiation cultures and analyzed by FACSAria on the basis of CD41 expression and forward/side scatter^low^. Mitochondria of MKs were stained with 200 nM MitoTracker Deep Red FM (Molecular Probes) at 37 °C for 30 min, followed by measuring the mean fluorescence intensity at 660 nm by flow cytometry. To analyze caspase-3 activation, platelets were isolated from mice and stained with allophycocyanin (APC)-conjugated anti-CD41 antibody (Biolegend) and FAM-DEVD-FMK (Cell Technology) that can enter the cells and irreversibly bind to activated caspase-3 specifically. The proportion of platelets with activated caspase-3 was quantified by the histogram at 488 nm according to the manufacturer’s instruction. All flow cytometric data were analyzed by FlowJo software (Tree Star).

### Proplatelet formation assay

CD41-positive mature MKs were sorted from bone marrow cells by FACSAria as described above and seeded in 24-well plates at a concentration of 2000 cells/mL in StemSpan serum-free expansion medium supplemented with 100 ng/mL TPO. After differentiation for 24 hr, the number of MKs displaying proplatelets relative to the total number of MKs was counted under a phase contrast microscope (Zeiss Axio Observer Z1) using a 40x objective at phase II.

### Luminescent assay of ATP and caspase-3/7 activity

Mature MKs were sorted from bone marrow cells and seeded in 24-well plate at 1 × 10^5^ cells per well in MK medium. At 30 minutes after LLLT, MKs were collected for ATP quantification using an ATP detection kit (Promega) according to manufacturer’s instructions. The ATP level was normalized to protein concentrations obtained by the Bradford method (BioRad Protein Assay kit). To measure caspase activation, platelets were prepared from blood samples of indicated mice, seeded in 96-well plate at 2 × 10^4^ platelets per well in MK medium, and incubated with or without anti-CD41 antibody for 2 hr, after which 100 μL of Caspase-Glo 3/7 reagents (Promega) were added in triplicate to each well 6 hr post-LLLT. After gently mixing in a plate shaker for 30 seconds and incubation at room temperature for 1 hr, luminescence of each sample was measured on a microplate reader (Molecular Devices) per the manufacturer’s instruction.

### Platelet preparation

Blood samples were collected from the retro-orbital venous plexus into polypropylene tubes (Axygen) containing 10% citrate-dextrose solution (Sigma). Platelet rich plasma was obtained by centrifugation of the blood samples at 200 g for 10 minutes. The resultant platelet rich plasma was then incubated with 0.1 μg/mL Prostaglandin E1 (Sigma) in PBS for 10 minutes at room temperature and concentrated by centrifugation at 2250 g for 15 minutes. The concentrated platelets were cultured *ex vivo* for 2 hr in the presence or absence of anti-CD41 antibody before subjecting to LLLT or sham light and measurement of caspase-3/7 activity by Caspase-Glo. To measure caspase-3 activation in platelets *in vivo*, platelets were prepared from mice after treated with or without anti-CD41 antibody for 2 days, along with two doses of LLLT or sham light. Caspase-3 activation was assayed by FAM-DEVD-FMK staining.

### Platelet lifespan analysis

Platelet lifespan was monitored by *in vivo* biotinylation as previously described^24^. In brief, 3 mg EZ-link-N-Hydroxysulfosuccinimide-Biotin (EZ-NHS-Biotin, Pierce) was intravenously injected into each mouse. Blood samples were obtained at indicated days and incubated with FITC-anti-CD41 and phycoerythrin (PE)-conjugated streptavidin (Biolegend), followed by flow cytometric analysis of biotinylated platelets.

### Statistical analysis

Statistical significance was assessed by two-tailed student’s *t* test for two-group comparison or one way analysis of variance (ANOVA) for multiple-group comparison using Graphpad Prism 6.0 (Graphpad Software). A value of P < 0.05 was considered statistical significance.

## Additional Information

**How to cite this article**: Yang, J. *et al*. Low-level light treatment ameliorates immune thrombocytopenia. *Sci. Rep.* **6**, 38238; doi: 10.1038/srep38238 (2016).

**Publisher’s note:** Springer Nature remains neutral with regard to jurisdictional claims in published maps and institutional affiliations.
